# Fluoroquinolone-Associated Tendinopathy Presenting As Calf Myotendinous Strain

**DOI:** 10.7759/cureus.92201

**Published:** 2025-09-13

**Authors:** Tristan S Moseley, Cameron Cluney, Lee A Dennis, Matthew D Overturf

**Affiliations:** 1 Medicine, Edward Via College of Osteopathic Medicine, Monroe, USA; 2 Anatomical Sciences, Edward Via College of Osteopathic Medicine, Monroe, USA

**Keywords:** achilles tendinopathy, adverse drug reaction, calf strain, fluoroquinolones, myotendinous junction

## Abstract

Fluoroquinolones are widely utilized antibiotics, but there is an increasing association with musculoskeletal complications, most notably Achilles tendon injury. We present the case of a 53-year-old woman with rheumatoid arthritis and a history of thyroid cancer who developed recurrent right calf pain and myotendinous strains approximately five months after sequential therapy with ciprofloxacin and levofloxacin. These episodes were localized to the gastrocnemius-soleus junction and occurred with minimal activity, without evidence of tendon rupture on MRI. Her condition was managed conservatively through physical therapy and activity modification; however, symptoms persisted with intermittent flares for over one year. This case underscores an atypical presentation of fluoroquinolone-associated tendinopathy, highlighting that such injury may extend beyond the Achilles tendon and manifest as recurrent myotendinous strains. Recognizing these less typical patterns is crucial, especially in patients with additional risk factors, and clinicians should exercise vigilance when prescribing fluoroquinolones or assessing patients with tendon-related symptoms.

## Introduction

Fluoroquinolones constitute a widely prescribed class of broad-spectrum antibiotics that function by inhibiting DNA gyrase and topoisomerase IV, enzymes crucial for bacterial replication [1]. Their oral bioavailability and extensive antimicrobial coverage render them valuable in the treatment of respiratory, urinary tract, gastrointestinal, and soft tissue infections [2]. Nonetheless, serious musculoskeletal complications have been increasingly acknowledged, notably tendinopathy and tendon rupture, predominantly affecting the Achilles tendon [3-4]. Risk factors encompass advanced age, concomitant corticosteroid therapy, renal impairment, and elevated physical activity [3,5]. While the precise mechanism remains incompletely understood, evidence indicates that oxidative stress, mitochondrial dysfunction, and extracellular matrix degradation may undermine tendon integrity [6-8]. Additional adverse effects include peripheral neuropathy, disturbances of the central nervous system, prolongation of the QT interval, and arthralgia [6,9]. Due to these concerns, the Food and Drug Administration (FDA) has issued advisories recommending the avoidance of fluoroquinolones in cases of non-life-threatening infections where safer alternatives are available [4,10]. This case study emphasizes recurrent calf myotendinous strain several months subsequent to fluoroquinolone administration, representing a less frequently reported manifestation of drug-related tendinopathy.

## Case presentation

**Figure 1:**
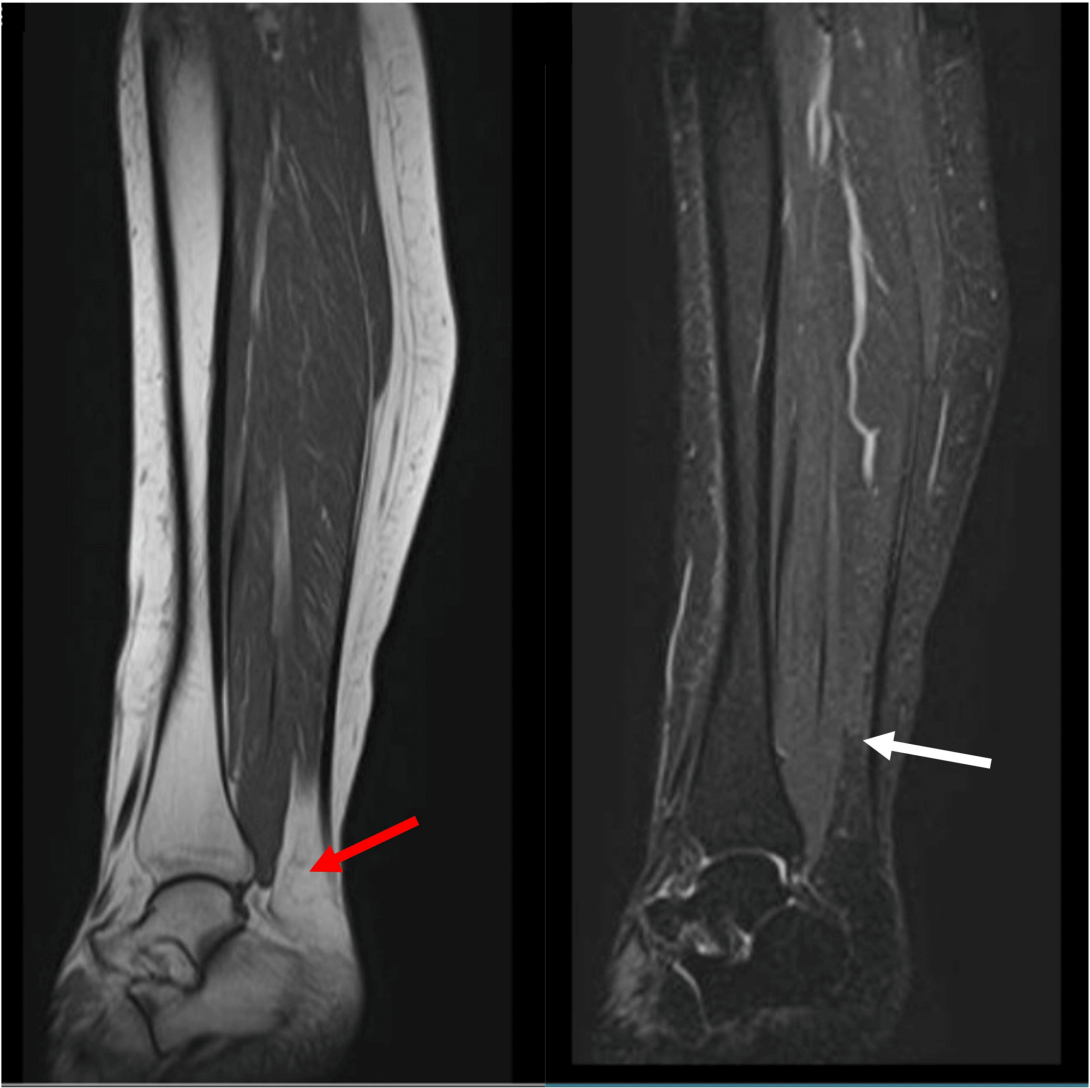
T1- and T2-weighted MRI of the left tibia Sagittal view T1 (left) and T2 (right) weighted MRI of the left tibia. The Achilles tendon (red arrow) appears intact on T1-weighted images, and there are no hyperintensities on T2-weighted images indicative of an inflammatory response within the myotendinous junction (white arrow).

A 53-year-old female with a medical history of rheumatoid arthritis and thyroid cancer presented with acute right calf pain following a sudden "pop" while playing pickleball. Initial examination suggested a gastrocnemius strain, which was managed conservatively. Over the subsequent months, she experienced a total of five similar episodes involving both lower extremities, each occurring during mild physical activity such as walking or stair climbing. The injuries are consistently localized to the musculotendinous junction of the calf muscles, sparing the Achilles tendon. Physical examinations during these episodes revealed focal tenderness and swelling at the gastrocnemius-soleus junction without signs of complete rupture. Magnetic resonance imaging (MRI) of the left tibia (Figure 1) demonstrated no evidence of acute tendon tears or ruptures. Patient history revealed completed sequential courses of ciprofloxacin and levofloxacin approximately five months prior to the onset of her initial injury. Given the pattern of recurrent strain localized to the musculotendinous junction, the absence of structural rupture, and prior exposure to fluoroquinolones, a diagnosis of fluoroquinolone-associated tendinopathy was considered. The patient was advised to refrain from future fluoroquinolone use and was referred for physical therapy focused on eccentric strengthening and the gradual reintroduction of activity. While her symptoms progressively improved, she continued to experience intermittent flare-ups with exertion more than one year after completing fluoroquinolone therapy. The persistent symptoms in both calves further support a systemic, drug-related process rather than an isolated mechanical injury.

## Discussion

This case exemplifies an atypical manifestation of fluoroquinolone-associated tendinopathy (Table 1). Although the Achilles tendon remains the most commonly reported site of pathology, the patient exhibited recurrent myotendinous junction strains involving both calves. Such localization underscores that fluoroquinolone-associated tendinopathy may encompass regions beyond the Achilles tendon, thereby emphasizing the importance for clinicians to be vigilant for a variety of presentations [3-4]. The absence of tendon rupture observed on MRI further indicates that structural imaging may not reliably exclude drug-induced tendon pathology [9]. Several factors likely contributed to the heightened susceptibility in this patient. Rheumatoid arthritis is recognized as an independent risk factor for tendon degeneration, and prior exposure to fluoroquinolones may have exacerbated this risk by impairing tendon cell metabolism and reparative processes [6-8]. The bilateral and recurrent pattern of injury, occurring months after discontinuation of the drug, reinforces evidence suggesting that fluoroquinolone toxicity can exert long-lasting effects on tendon integrity [9]. Importantly, even mild physical activity was sufficient to induce recurrent strains, consistent with previous observations that drug-related tendinopathy is not confined to high-stress exertion [3,5].

**Table 1:** Comparison of reported fluoroquinolone-induced tendinopathy cases vs. the present case

Feature	Previously reported cases	Present case (atypical)
Typical tendon involved	Predominantly Achilles tendon (>90%) involvement [1]	Musculotendinous junction of the gastrocnemius – rarely reported
Onset timing	Usually within days to weeks after starting fluoroquinolone therapy	Five months following fluoroquinolone treatment
Risk factors	Older age, renal impairment, corticosteroid use, transplantation, high activity level	Middle-aged, rheumatoid arthritis, mild physical activity
Clinical presentation	Acute pain, swelling, tenderness, sometimes rupture	Focal tenderness and swelling at the gastrocnemius-soleus junction
Diagnostic findings	Ultrasound/MRI typically showing Achilles involvement	MRI demonstrated no Achilles involvement
Management	Immediate discontinuation of fluoroquinolone; conservative measures; surgery if ruptured	Physical therapy focused on eccentric strengthening, gradual reintroduction of activity, refrain from future fluoroquinolone treatment
Outcome	Variable; recovery over weeks–months, though ruptures may lead to chronic disability	Symptoms improved, intermittent flare-ups following exertion more than one year following fluoroquinolone treatment
Novelty highlight	Cases cluster around Achilles tendinopathy with classic risk profile	First report of myotendinous junction of calf muscles, expands spectrum of fluoroquinolone-induced tendinopathy

From a clinical perspective, this case highlights the importance of meticulous medication history-taking in patients presenting with tendon or myotendinous pain. The identification of fluoroquinolone-associated tendinopathy should prompt immediate discontinuation of the offending agent and the avoidance of subsequent exposure [3,10]. Conservative treatment strategies, including eccentric strengthening exercises and activity modifications, can facilitate functional recovery; however, symptoms may persist for more than one year, as observed in this case. The presentation of bilateral, recurrent, and non-Achilles involvement broadens the clinical spectrum of fluoroquinolone-associated tendinopathy and emphasizes the necessity for vigilance in recognizing these atypical manifestations.

## Conclusions

This case broadens the clinical understanding of fluoroquinolone-associated tendon injury by illustrating recurrent right calf myotendinous strains in the absence of Achilles involvement or structural rupture. The delayed onset, bilateral distribution, and persistence of symptoms highlight the systemic and enduring effects of fluoroquinolone toxicity on tendon integrity. Healthcare professionals should diligently review medication histories in patients presenting with tendon or myotendinous pain, as early identification and discontinuation of fluoroquinolones are vital in preventing additional morbidity. Ultimately, this case emphasizes the importance of heightened clinical awareness regarding atypical presentations of fluoroquinolone-associated tendinopathy and advocates for continued caution when prescribing these agents, especially when alternative, safer options are accessible.

## References

[1] Khaliq Y, Zhanel GG (2003). Fluoroquinolone-associated tendinopathy: a critical review of the literature. Clin Infect Dis.

[2] Kim GK (2010). The risk of fluoroquinolone-induced tendinopathy and tendon rupture: what does the clinician need to know?. J Clin Aesthet Dermatol.

[3] Lewis T, Cook J (2014). Fluoroquinolones and tendinopathy: a guide for athletes and sports clinicians and a systematic review of the literature. J Athl Train.

[4] Shu Y, Zhang Q, He X, Liu Y, Wu P, Chen L (2022). Fluoroquinolone-associated suspected tendonitis and tendon rupture: a pharmacovigilance analysis from 2016 to 2021 based on the FAERS database. Front Pharmacol.

[5] Morales DR, Slattery J, Pacurariu A, Pinheiro L, McGettigan P, Kurz X (2019). Relative and absolute risk of tendon rupture with fluoroquinolone and concomitant fluoroquinolone/corticosteroid therapy: population-based nested case-control study. Clin Drug Investig.

[6] Simonin MA, Gegout-Pottie P, Minn A, Gillet P, Netter P, Terlain B (2000). Pefloxacin-induced achilles tendon toxicity in rodents: biochemical changes in proteoglycan synthesis and oxidative damage to collagen. Antimicrob Agents Chemother.

[7] Kaleagasioglu F, Olcay E (2012). Fluoroquinolone-induced tendinopathy: etiology and preventive measures. Tohoku J Exp Med.

[8] Arabyat RM, Raisch DW, McKoy JM, Bennett CL (2015). Fluoroquinolone-associated tendon-rupture: a summary of reports in the Food and Drug Administration's adverse event reporting system. Expert Opin Drug Saf.

[9] Tsai WC, Yang YM (2011). Fluoroquinolone-associated tendinopathy. Chang Gung Med J.

[10] Zabraniecki L, Negrier I, Vergne P, Arnaud M, Bonnet C, Bertin P, Treves R (1996). Fluoroquinolone induced tendinopathy: report of 6 cases. J Rheumatol.

